# Advances in Long Term Physical Behaviour Monitoring

**DOI:** 10.1155/2016/6745760

**Published:** 2016-04-11

**Authors:** Jorunn L. Helbostad, Lorenzo Chiari, Sebastien Chastin, Kamiar Aminian

**Affiliations:** ^1^Department of Neuroscience, Faculty of Medicine, University of Science and Technology, 7491 Trondheim, Norway; ^2^Department of Electrical, Electronic, and Information Engineering, University of Bologna, 40136 Bologna, Italy; ^3^School of Health and Life Sciences, Caledonian University, Glasgow G4 0BA, UK; ^4^École Polytechnique Fédérale de Lausanne, 1015 Lausanne, Switzerland

Along with the development of cheap and easily available body-worn and environmental sensors, monitoring of physical behaviour in everyday life situations is now possible and has become increasingly popular in research and for clinical purposes. Availability of such sensors and instruments may move assessment of physical function and activity from controlled laboratory settings to the natural environments and situations where people live their daily lives. It may also shift focus of the assessment from what people are capable of doing, as typically assessed in lab, to what people actually do and how they do it in their daily lives. Availability of a new generation of sensing technologies gives new opportunities for gaining knowledge with regard to health and function, but it also raises several challenges! One of the current challenges is lack of standards for data collection and processing, making comparison and harmonisation of data across studies and systems limited [1].

Body-worn sensors may include accelerometers, gyroscopes, magnetometers, barometers, light sensors, and global positioning systems (GPS) and are used for a range of different purposes like assessing the amount and patterns of physical activity and related energy expenditure, sleep pattern, and movement characteristics of specific activities, for example, gait and rising from a chair or fall detection. Such information may further be used to develop risk assessment tools for diseases, functional decline, and falls and for giving individualised feedback on physical behaviour as part of a preventive intervention. In a home setting, environmental sensors, like cameras, radars, infrared light sensors like the Kinect system, or even optic fibres embedded in the flooring, are available for monitoring behaviours like mobility and movement patterns, falls, sleep, and sedentariness as well as exercise behaviour while playing exergames.

Even if the technology is easily available, the understanding of the signals derived from the monitoring still needs more attention, and algorithms developed for different purposes and settings need more thorough testing for reliability, validity, and sensitivity to change [2]. Furthermore, in order to motivate people to adopt the technology, its utility has to be linked to what people need and want to know about themselves and what is needed in order to prevent or treat diseases [3]. Moreover, the technology must be unobtrusive, and usability has to be in focus when developing the systems [4]. Mobile technology commonly used by people, like smartphones [5] and smartwatches, may increase adherence to the use of the technology also for monitoring purposes.

In this special issue, we have solicited submission of research papers applying monitoring technology that can stimulate the continuing efforts to better understand physical behaviour as part of preventive health care and rehabilitation. The six papers that are included demonstrate usage of a variety of monitoring technologies applied in different populations and for different purposes, including assessment of gait characteristics related to fall risk, heart rate variability in relation to chronic neck pain, differences between physical performance and free-living activity in older people, quantification of outdoor mobility in older people, assessment of cardiometabolic risk and health-related quality of life, and in-home assessment of risk of falling in people with Parkinson's disease. The papers nicely demonstrate the current state of the research field, by focusing either on development of new features to describe free-living physical behaviour or on applying the technology to understand aspects of behaviour that has not been easily assessable previously.

The European population is ageing and more people live with chronic diseases, while at the same time the number of employees per pensioner is decreasing. Technology and its applications presented in this special issue might be of importance for solving some of these challenges by making people able to monitor and control their own health and function, thereby staying independent longer and reducing health care costs. The field of mobile health technology (mHealth) and telemedicine is moving forward at a high speed, but there is still a gap between development of new methods and what is implemented in clinical practice. Clinical intervention studies with sufficient sample sizes will be needed in the near future to demonstrate feasibility and added value of using the technology with respect to usual standard of care provided in our health care systems.

*Jorunn L. Helbostad* *Lorenzo Chiari* *Sebastien Chastin* *Kamiar Aminian*
